# Sex, health status and habitat alter the community composition and assembly processes of symbiotic bacteria in captive frogs

**DOI:** 10.1186/s12866-023-03150-y

**Published:** 2024-01-23

**Authors:** Senlin Liu, Sewar Imad, Sarfraz Hussain, Shuiqing Xiao, Xiaowei Yu, Hui Cao

**Affiliations:** 1 College of Life Sciences/Key Laboratory of Agricultural Environmental Microbiology, Ministry of Agriculture and Rural Affair, Nanjing Agricultural University, 6 Tongwei Road, Nanjing, Jiangsu, 210095 People’s Republic of China; 2 School of Life Sciences, University of Warwick, Gibbet Hill Road, Coventry, UK; 3 Jiangxi Normal University, Nanchang, JX China

**Keywords:** Frog, Sex, Health status, Habitat, Network structure, Assembly

## Abstract

**Background:**

Frogs are critical economic animals essential to agricultural ecosystem equilibrium. However, Meningitis-like Infectious Disease (MID) often affects them in agricultural settings. While frog-associated microbiota contribute to elemental cycling and immunity, the effects of frog sex and health on gut bacteria remain understudied, and the relationship between frog habitat and soil microbes is unclear. We aimed to determine how frog sex, health status and habitat influence symbiotic bacteria and community assembly mechanism to provide guidance for sustainable frog farming and conservation.

**Results:**

We employed 16S rRNA sequencing to investigate gut microbiota differences in relation to frog sex and health status. We also compared symbiotic communities in frog-aggregation, native and soybean soil on the farm. Results showed that gut bacterial β-diversity and taxonomy were markedly influenced by frog sex and health. Healthy frogs had more robust gut bacterial metabolism than frogs infected with MID. Cooccurrence network analysis revealed that healthy female frogs had more complex microbial network structure than males; however, diseased males showed the greatest network complexity. The assembly mechanism of gut bacteria in male frogs was dominated by deterministic processes, whereas in female frogs it was dominated by stochastic processes. Among symbiotic bacteria in frog habitat soils, deterministic processes predominantly shaped the community assembly of soybean soil. In particular, soybean soil was enriched in pathogens and nitrogen functions, whereas frog-aggregation soil was markedly increased in sulphur respiration and hydrocarbon degradation.

**Conclusion:**

Our study reveals that sex mainly alters the interaction network and assembly mechanism of frog intestinal bacteria; MID infection significantly inhibits the metabolic functions of intestinal bacteria. Furthermore, diverse frog habitat soils could shape more symbiotic bacteria to benefit frog farming. Our findings provide new horizons for symbiotic bacteria among frogs, which could contribute to sustainable agriculture and ecological balance.

**Supplementary Information:**

The online version contains supplementary material available at 10.1186/s12866-023-03150-y.

## Introduction

In recent years, the study of symbiotic microbial communities in amphibians, particularly frog gut microbiota, has gained interest due to their potential impacts on host health and ecological interactions. Frogs have evolved in environments surrounded by bacteria, forming highly complex symbiotic relationships [1]. Gut microbes play vital roles in host nutrient metabolism, disease resistance, immunity, and overall health by participating in nutrient metabolism and pathogen defense [2, 3]. The black-spotted frog (*Pelophylax nigromaculata*), an amphibian from the order Anura and genus *Pelophylax*, is crucial for maintaining agricultural ecosystem balance [4]. However, human activities and climate change have raised concerns about the survival of *P. nigromaculata* [5]. Factors such as life stage, sex, diet, habitat conditions, seasonal variation, and host genetics influence gut microbiota composition and diversity [6–8]. For example, habitat degradation and anthropogenic disturbances can alter gut microbial community structure, potentially affecting amphibian health [9]. Symbiotic bacterial community assembly is crucial for host adaptation to changing environments [10]. Jin Zhou found that urbanization increased the stochasticity of microbial communities in frogs and reduced their ecological stability [11]. Seasonal shifts led to decreased frog microbial network complexity, while deterministic processes increased bacterial assembly from summer to fall [12]. Investigating these factors is essential for understanding the ecological role of symbiotic microbes in amphibians and informing conservation strategies.

Sex plays a pivotal role in shaping the composition of gut microbiota [13], yet the underlying mechanisms remain elusive. Meijer et al. investigated the presence of bacterial communities, such as *Alistipes* and *Rikenella*, in germ-free male mice, which proliferated in the absence of innate immune defenses. Upon transfer to germ-free female mice, these bacterial communities induced weight loss and inflammation [14]. Furthermore, Markle et al. demonstrated that male mice exhibited increased testosterone levels, promoting the growth of specific gut bacteria that protect against the development of type 1 diabetes [15]. In amphibian populations, females tend to be larger than males. According to the optimal foraging theory, larger frogs are likely to consume larger prey, consequently affecting their gut microbiota [16]. Research on frogs has indicated that although bacterial diversity did not significantly differ between sexes, community composition below the class level could reflect sex differences, particularly concerning Enterobacteriales, Enterobacteriaceae, and Peptostreptococcaceae [17]. These studies have elucidated the influence of sex on the composition and potential functionality of frog gut microbiota. However, limited research exists on the relationship between sex and gut microbial communities in black-spotted frogs, particularly regarding the complex interactions and assembly processes within these communities.

Amphibian health has drawn notable attention recently due to their susceptibility to environmental shifts and emerging diseases. The gut microbiota acts as a crucial immune organ in amphibians [18]. Kamada et al. identified two primary strategies through which gut microbiota help hosts resist pathogen invasion: competing for limited nutrients and modulating host immune responses [19]. In addition, Kruger et al. found variations in the skin microbiota of Brazilian frogs depending on host species and location, with no significant differences between Bd-infected and healthy individuals [20]. This suggests that changes in the bacterial composition of the frog gut may reflect host species and environmental factors rather than health status. Beneficial gut symbionts, such as *Janthinobacterium* [21] and *Akkermansia* [22], actively promote amphibian resistance to foreign pathogens. Meningitis-like infectious diseases (MID), also known as frog cataract and tarsal maggot, may be related to changes in gut microbial communities, but research remains limited [23]. Wengang Li reported that compared to their healthy counterparts, bullfrogs infected with MID had higher oral and intestinal microbial richness and abundance, and the abundance of *Elizabethkingia* increased while *lactococci* decreased [23]. Despite these advancements, knowledge about the response of frog symbiotic microbiota to health conditions remains limited.

Soil microorganisms play a crucial role in the soil environment, participating in processes such as mineralization of organic matter, formation and decomposition of humus, and transformation of nutrient elements [24]. Various frog habitats exist in breeding farms, including native soil, soybean soil, and frog-inhabited soil, necessitating the investigation of the relationship between soil microbes and frogs. Studies have shown that soybean cultivation has a more pronounced effect on the composition of rhizobia in agricultural soils compared to native soils, thereby reducing the complexity of microbial community interactions [25]. Elly proposed that during the natural restoration process of fallow agricultural land, soil biotic community composition changes, networks contract, and carbon sequestration efficiency increases significantly [26]. To reduce the use of fertilizers and pesticides, the rice-frog ecosystem has emerged; researchers found that rice-frog (RF) cultivation significantly enriched the rhizosphere microbial communities of *Sandaracinaceae*, *Anaerolineaceae*, and *Candidatus Nitrososphaera*, which may be involved in improving nutrient cycling and promoting plant growth [27]. However, research on the response of soil bacteria in different land use types to the introduction of frogs remains scarce.

In this study, we focused on *P. nigromaculata* from intensive frog farms in southern China, where severe MID infections are prevalent. We analyzed the effects of sex and health status on gut microbial communities using 16S rRNA amplicon sequencing. Additionally, we collected samples from frog-aggregation soil (AS), native soil (NS), and soybean soil (SS) in the farms to investigate variations in soil microbiota. We aimed to address three questions: (1) whether frog sex influences gut microbiota; (2) the variations in gut microbial communities and potential metabolic functions related to frog health status; and (3) the responses of soil bacterial communities to frog habitats, as well as the bacterial network structure and community assembly patterns. Research on frog microbiomes is crucial for maintaining host health and conserving amphibian habitats. This study has the potential to refine frog farming practices, improve the health of farmed frogs and provide a basis for intensive frog rearing. Moreover, it may contribute to the promotion of effective, sustainable amphibian conservation strategies.

## Materials and methods

### Gut and soil samples collection

Sixteen frogs (*P. nigromaculata*), consisting of eight females and eight males, were collected from Xuanzhou District, Anhui Province, South China (30°50’8.4"N, 118°36’9"E) in February 2020 (Supplementary Fig. S1). Frog samples were selected according to the following criteria: (1) all samples were from the same rearing area; (2) adult frogs were 7 months old; (3) rearing conditions were the same as before collection. Each frog was individually placed in a plastic container and transported to the research laboratory for further analysis. To prevent bacterial contamination of samples, forceps and scissors were sterilized using autoclave and high-intensity UV light source before the frogs were sacrificed. Frogs were euthanized immediately using 1% aqueous solution of tricaine methanesulfonate (MS-222, Sigma-Aldrich). Before processing, the euthanised frogs were checked for cessation of heartbeat to confirm their death. Following the procedure described by Mashoof et al. [28], the frogs were first rinsed with tap water and then rinsed with sterile water, and the intestinal contents from the stomach (excluding the stomach contents) to the anal intestinal contents were collected within 20 min after frog euthanasia; then placed in sterilised EP tubes and stored at -80 °C for later analysis. At necropsy, some frogs were found to be infected with MID and were classified as unhealthy. Based on sex and health status, they were labelled MH (male healthy, n = 4), FH (female healthy, n = 4), MNH (male unhealthy, n = 4) and FNH (female unhealthy, n = 4). To investigate the effect of sex on gut microbiota, M group (male, n = 8) and F group (female, n = 8) were compared. To evaluate the effect of health status on gut microbiota, infected individuals were assigned to the NH group (n = 8) and uninfected individuals were assigned to the H group (n = 8).

To investigate the adaptation of frog symbiotic microbes to external habitats, we collected soil from the same frog breeding area, including three soil environment types: (1) Native soil (NS) was collected from loose soil near the frog breeding site, where fewer frogs were present and active; (2) Frog-aggregation soil (AS) was collected from the bottom of the tray where frogs prefer to congregate, characterized as consistently moist, dark, and isolated from other environmental influences; (3) Soybean soil (SS) was collected from areas where soybean plants are grown, providing shelter and food for the frogs and creating a unique habitat. Each of these three soil types contained three replicates (using the multi-point mixed sampling method) [29], for a total of nine samples. Soil samples were collected from the top layer (0–20 cm) after removing the surface grass and approximately 10 g of each soil sample was collected from each control pool and immersed in 50 ml of LifeGuard solution. All samples were stored at -20 °C until required.

### Total microbial DNA extraction, PCR amplification and illumina sequencing

According to the manufacturer’s instructions, Omega’s Environmental DNA Extraction Kit was used to extract microbial DNA from frog gut and soil samples. The universal primer combination F338 (5’-ACTCCTACGGGAGGCAGCA-3’) and R806 (5’-GGACTACVSGGGTATCTAAT-3’) amplified the V3-V4 regions of the 16 S rRNA gene [30]. PCR thermocycling consisted of 95 °C for 5 min, 30 cycles of 30 s at 95 °C, 50 and 72 °C, and a final extension at 72 °C for 5 min. Prior to ligation of the Illumina barcodes and adaptors, PCR products were purified using the Omega e.Z.N.A. TM CyclePure Kit, measured and aggregated in equimolar proportions. The libraries were sequenced according to the Illumina MiSeq instructions.

### Microbiome bioinformatics and statistical analysis

QIIME2 (version QIIME2-2022.2) was employed to process the raw sequence data [31]. Paired reads (2 × 250 bp paired-end mode) from HiSeq4000 platforms were demultiplexed, filtered using vsearch, and subjected to quality control as follows: sequences with a length of 200 bp or an average quality score of 25 were eliminated, and ambiguous bases were not permitted. High-quality reads (> 97% identity) were clustered into operational taxonomic units (ASVs) using vsearch cluster-features-de-novo [32]. Samples were rarefied to the same sequence depth (33,957 bacterial sequences per sample), and clustered feature tables were further filtered using QIIME2 feature-table filter-features (0.001%) [33]. Taxonomy was assigned to ASVs using the Silva v138 database and the Naive Bayes classifier [34]. After removing chloroplast and mitochondrial sequences, the final dataset comprised 1,214 ASVs for further analysis. The NCBI Sequence Read Archive (SRA) accession number for the genomic sequencing data is PRJNA1023626.

### Co-occurrence network analysis measures of diversity and community structure

We utilized R v4.1.3.1 for data analysis and visualization. We assessed group differences through α diversity indices (Chao1, Shannon, Simpson, and Phylogenetic Diversity) using One-way ANOVA and Tukey HSD tests (p < 0.05) [35]. The β diversity was evaluated using PERMANOVA, ANOSIM (with 999 permutations), and Nonmetric multidimensional scaling (NMDS), as implemented in the R ‘vegan’ package [36]. To investigate the abundance of phyla and genera in amphibian intestine and soil bacteria, we generated bar charts and CIRCOS plots [37]. Wilcoxon signed-rank tests with Bonferroni corrections were performed between F and M, and between H and NH samples at the genus level; samples labeled by frog habitats (AS, NS, SS) were analyzed using the Kruskal-Wallis rank sum test.

We constructed co-occurrence networks of gut bacteria using the ‘WGCNA’ R package, based on Spearman’s correlation coefficients (r > |0.9|, p < 0.01) [38]. For soil bacterial networks, we identified significant associations (r > |0.9|, p < 0.01) among 50 major genera using Spearman’s correlation tests. We visualized these networks and calculated their properties using Gephi 0.92 software [39]. We evaluated variations in network structure among frog groups by measuring the number of nodes, number of edges, average degree, degree centralization, graph density, graph modularity, and betweenness centralization. We identified the putative role of each node using two network topological features, intra-module connectivity (Zi) and inter-module connectivity (Pi): network hubs (Zi > 8 and Pi > 0.62) [40]. Keystone taxa, or network hubs, are species that may help maintain microbial community structure [41].

We then used PICRUSt to predict the microbial functions of the frog gut samples [42]. We used the MetaCyc databases and seeded them with 16 S rRNA gene sequences to generate a bar graph showing microbial functional profiles. We detected significant differences in MetaCyc pathways (level 2) among amphibian symbionts using an equal variance t-test [43]. Additionally, we performed functional annotation of soil bacteria using the “functional annotation of prokaryotic taxa” (FAPROTAX) program [44], which allowed for a comprehensive understanding of the functional roles of these bacterial communities.

### The calculation of community assembly process

We assessed the assembly processes of bacterial communities in the samples by calculating the Beta Nearest Taxon Index (βNTI). Utilizing the ‘comdist’ function available in Phylocom v4.2 as part of the ‘picante’ package, we determined the β-mean nearest taxon distance (βMNTD) deviation from the null model through βNTI values. Based on the findings by Stegen et al. [45], when |βNTI| exceeded 2, deterministic processes were the primary drivers of the microbial community. In contrast, when βNTI values were situated between − 2 and + 2, stochastic processes predominantly shaped the microbial community structure [46]. To evaluate pairwise microbial community turnover and further characterize assembly processes, we applied the Raup-Crick metric (RCbray). The results indicated that community assembly were subject to the influence of dispersal limitation (|βNTI| < 2 and RCbray > + 0.95) and homogenizing dispersal (|βNTI| < 2 and RCbray < -0.95) [47].

## Results

### Sequencing depth, alpha and beta diversity of captive frogs and soil microbiota

**Fig. 1:**
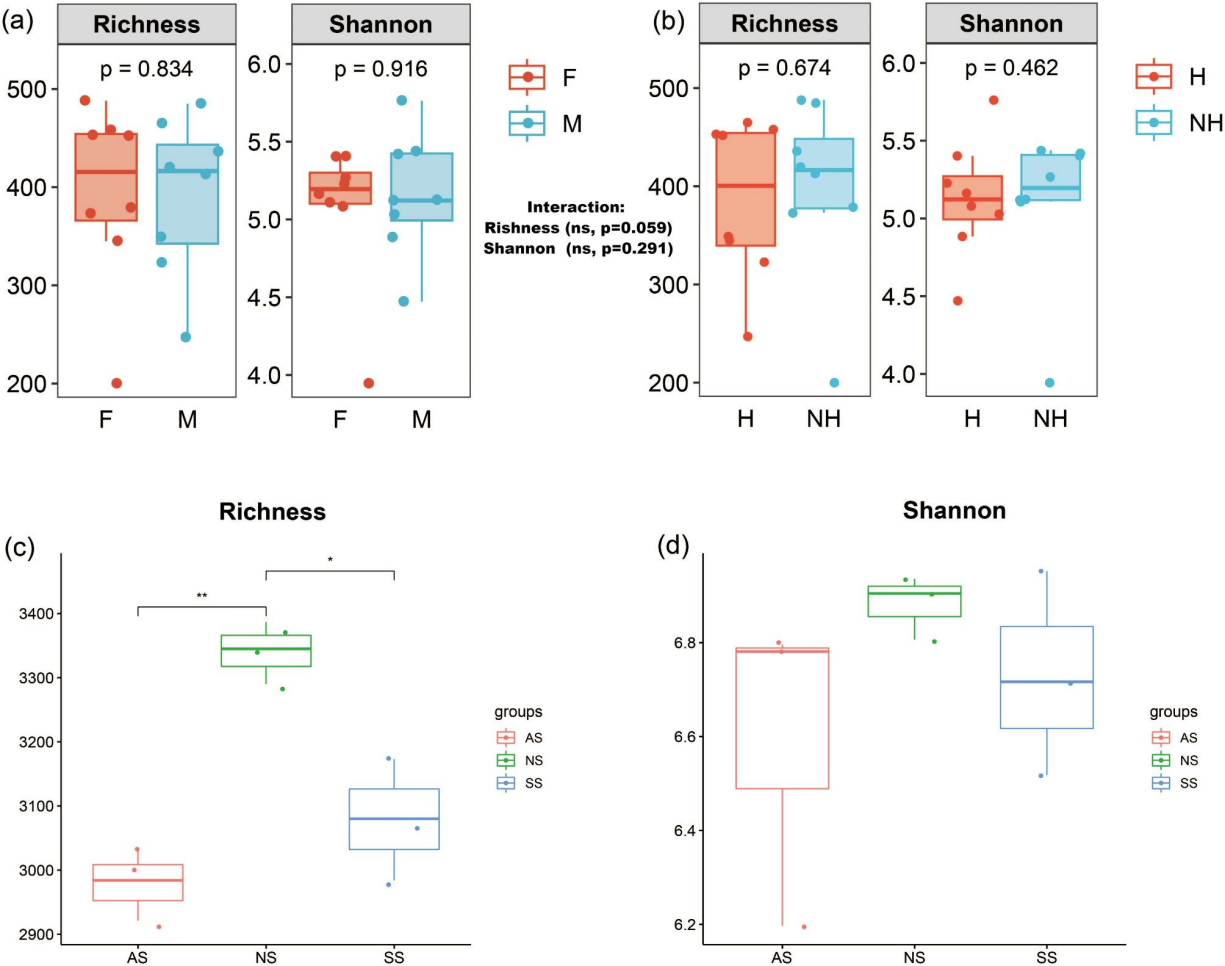
Displays α-diversity comparisons between female and male frogs **(a)** and among health states **(b)** using t-tests. Two-way ANOVA detected sex and health interaction differences, indicated by different capital letters. Richness **(c)** and Shannon index **(d)** comparisons between frog habitat soils (AS, NS, SS) are marked: * indicates p < 0.05 and ** indicates p < 0.01 significance; ‘ns’ indicates no significance at p = 0.05 level

The effect of sex and health status on the gut microbiota composition in captive frog samples was investigated. A 16S rRNA microbial data set consisting of 1,018,720 filtered high-quality sequences was generated, with an average of 63,670 ± 6,868 sequences per frog sample (Additional file 2). A total of 1,214 microbial species (ASVs) were identified in the gut communities based on > 97% sequence similarity, with an average length of 437 bp per sequence. When assessing microbial α-diversity, there were no significant differences in overall bacterial diversity among the four frog groups in terms of bacterial richness, Shannon index or phylogenetic diversity (Fig. S2). Similarly, neither the distinction between healthy and unhealthy frogs nor between females and males showed significant differences in richness or Shannon index (t-test, p > 0.05) (Fig. 1a-b). The interaction of sex and health status did not influence the diversity or abundance of the bacterial communities in the frog gut (two-way ANOVA, p > 0.05). However, when comparing α-diversity between the host environment and the gut, soil microbes showed increased diversity (Fig. 1c-d). This can be attributed to the combined effects of host species and extrinsic abiotic environmental elements. In contrast, soil bacteria contained 4,461 ASVs. Our examination of the bacterial alpha indices revealed remarkable differences in frog habitats only for community richness (Chao1). Chao1 of soil bacteria in different frog habitats showed that NS was significantly increased (t-test, p = 0.01) compared to AS and SS.

**Fig. 2:**
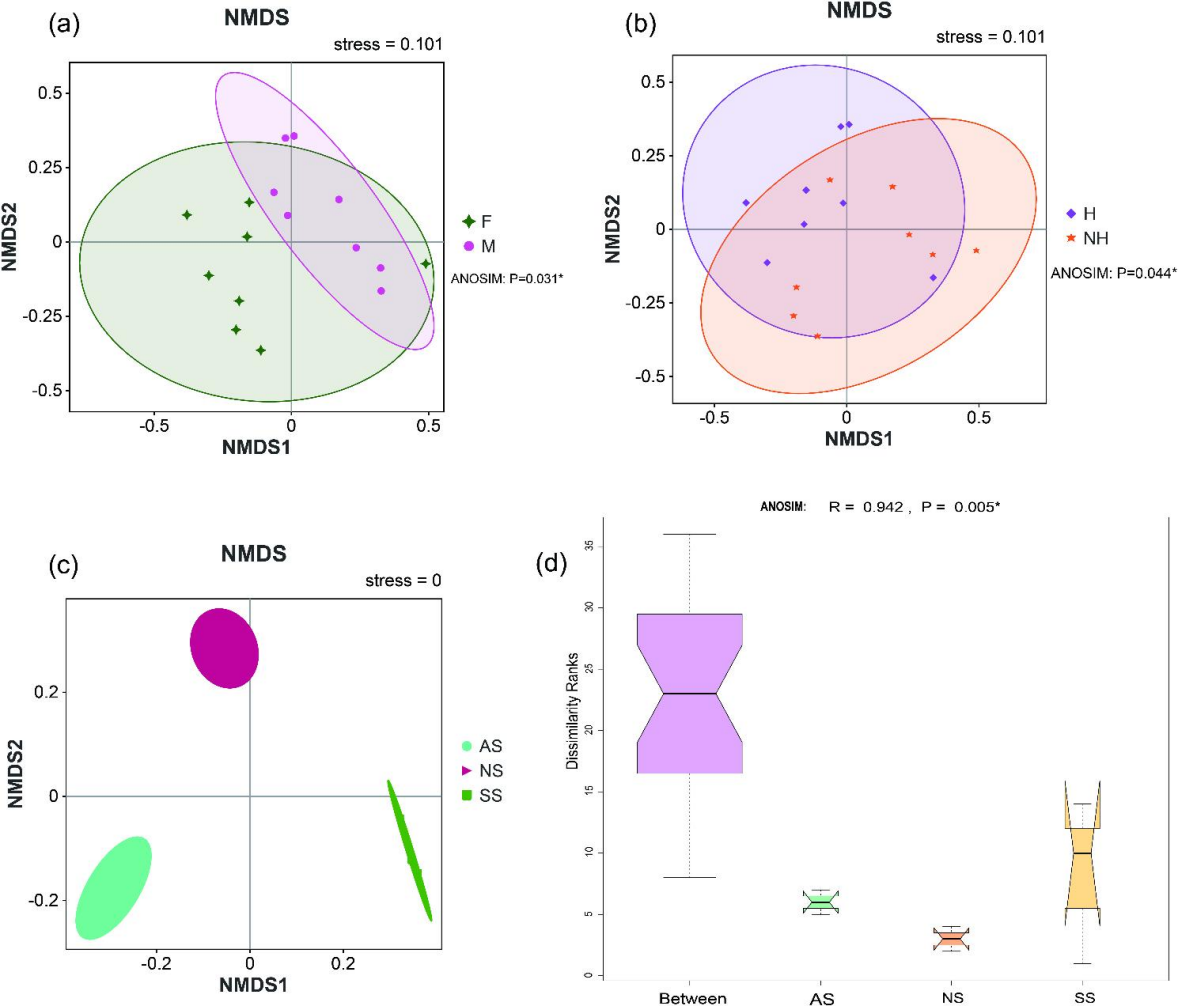
β-Diversity of bacterial community structure in frog gut and soil: **(a)** Nonmetric Multidimensional Scaling (NMDS) plot illustrating gut bacterial communities in frogs, comparing F (female) and M (male). **(b)** Comparison of gut microbiota in frogs between H (Healthy) and NH (Unhealthy). **(c)** Grouping of samples by frog habitats (AS, NS, SS), with each dot representing each sample. **(d)** Results displayed as relative variable importance (R) and significance (p) calculated using PERMANOVA (ANOSIM)

The NMDS plot (Fig. 2a) showed that the bacterial communities segregated significantly (ANOSIM: Bray-Curtis, r = 0.650, p = 0.031) into two major groups, the F group and the M group. According to the Bray-Curtis dissimilarity matrix (ANOSIM: Bray-Curtis, r = 0.740, p = 0.044) (Fig. 2b), the gut microbiota composition of captive amphibians from the H and NH groups was significantly different. In addition, soil samples were separated and significant differences in bacterial composition were observed between frog habitats (r = 0.942, p = 0.005, Fig. 2c-d). It clearly shows the difference in bacterial diversity in the two symbiotic (gut and soil) microbiomes of the frog.

### Compositional and distributional patterns of gut bacteria, habitat soils related with frogs

**Fig. 3:**
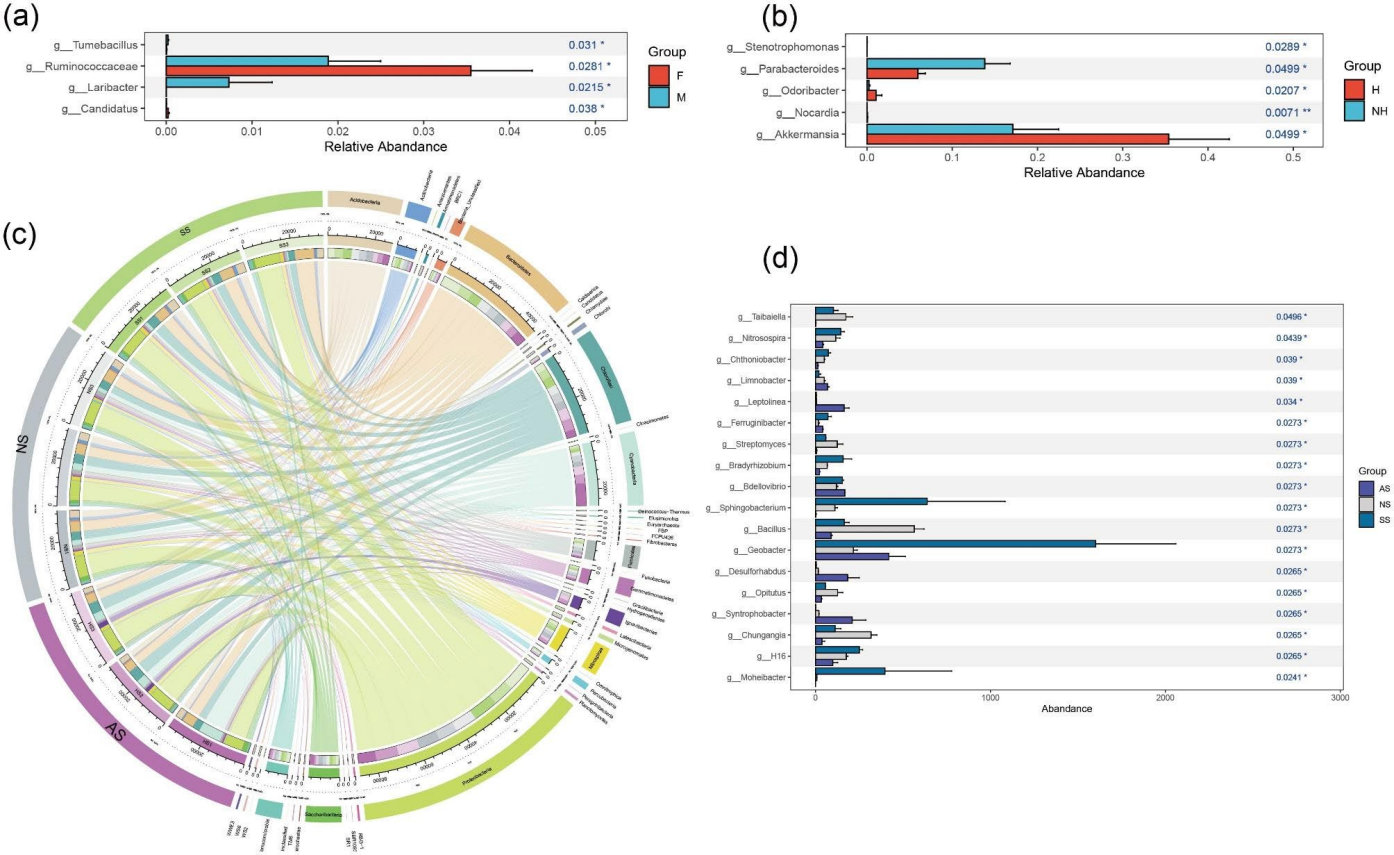
Differential abundance of bacterial genera in frog guts and soils: **(a)** Wilcoxon signed-rank tests with Bonferroni corrections were conducted between F (female) and M (male) frog samples at the genus level. **(b)** Comparison of gut bacterial communities in frogs between H (Healthy) and NH (Unhealthy). **(c)** CIRCOS plots depict the relative abundance of soil bacteria at the phylum level. **(d)** Samples grouped by frog habitats (AS, NS, SS) were analyzed using Kruskal-Wallis rank sum tests. Only differentially abundant genera are displayed, and asterisks indicate significant differences between groups

Taxonomic assignment analysis revealed 10 phyla in captive frogs, and the most abundant phyla in all frogs were Firmicutes, Bacteroidetes and Proteobacteria (Fig. S3). At the phylum level, there were no significant differences in relative abundance between sexes or health status groups (p > 0.05). Among the habitat soils, the CIRCOS plots showed that 14 phyla were dominant (> 1%), including Proteobacteria, Bacteroidetes, Acidobacteria, Firmicutes, Nitrospirae, Saccharibacteria, Verrucomicrobia, Actinobacteria, Chloroflexi and others. Further comparison showed that Firmicutes was the most abundant in NS, Ignavibacteriae was much more abundant in AS than in other soils, and Actinobacteria occupied the highest relative abundance in SS. However, there was no significant difference between the soil microbiota at the phylum level (Fig. 3c).

At the genus level, frog gut bacteria contained 21 dominant genera, and *Bacteroides* (10.6%) was the most dominant bacterial genus, followed by *Citrobacter* (6.6%), *Parabacteroides* (5.3%), and *Akkermansia* (4.90%) (Fig. S4). Furthermore, our result showed that *Ruminococcaceae* was significantly enriched in the female group, whereas *Laribacter* was significantly enriched in the male group (Kruskal-Wallis rank sum test, p < 0.05; Fig. 3a) among the top 100 genera. Five genera were significantly different between two health statuses, with *Parabacteroides* significantly higher in NH than in H, whereas *Odoribacter* and *Akkermansia* were significantly higher in H than in NH (Fig. 3b). Among the habitat soils, there were 18 genera among all 223 identified genera that differed significantly among soil groups, among which *Bacillus, Nitrosospira*, and *Geobacter* were among the dominant genera (Fig. S5), with *Bacillus* being significantly higher in NS than in other groups. *Geobacter*, *Sphingobacterium, Bradyrhizobium* and *Moheibacter* were significantly higher in relative abundance in SS than in other soils (Fig. 3d).

### Potential cooccurrence patterns of bacterial community in frog gut and habitat soils

**Fig. 4:**
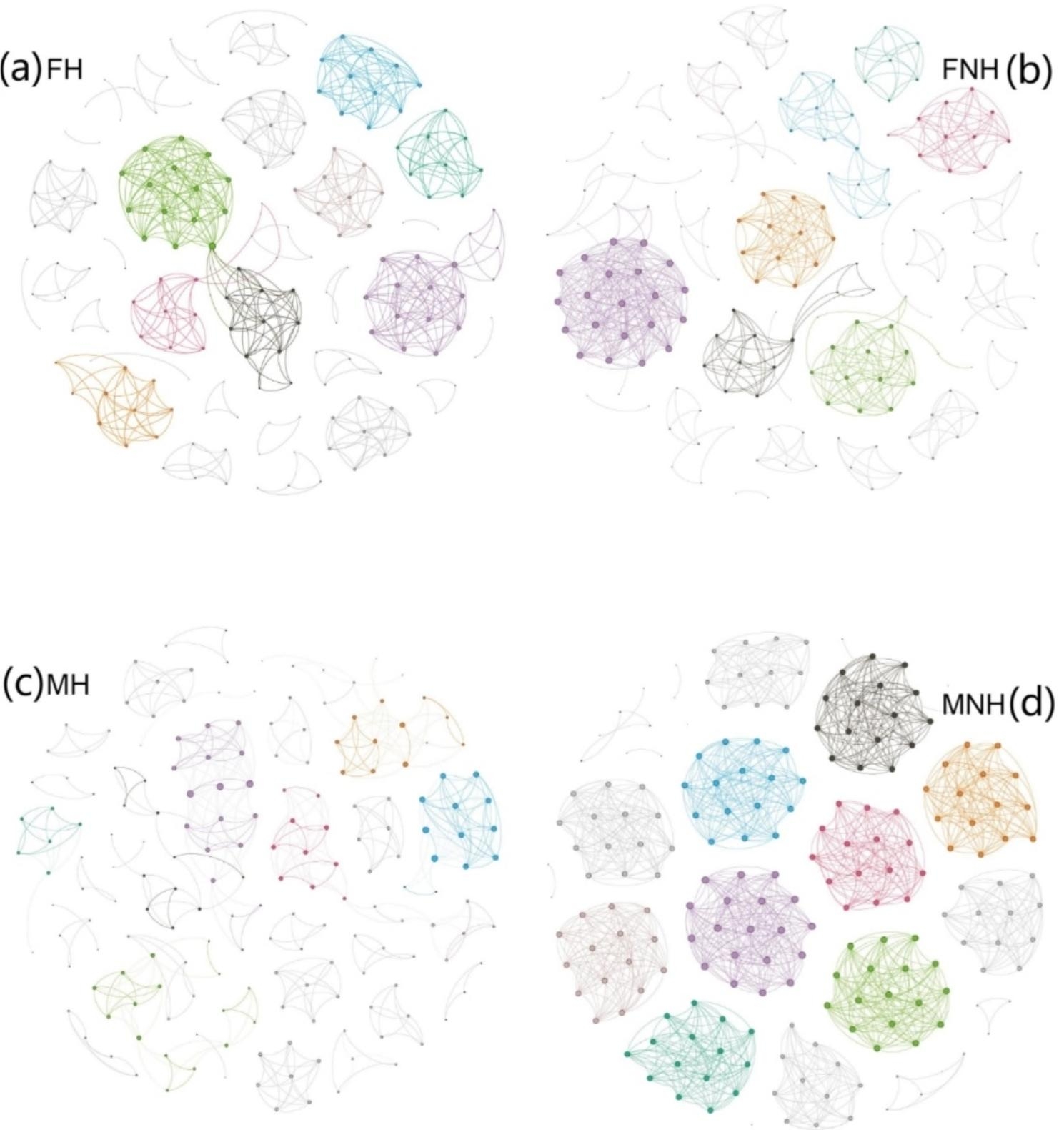
Co-occurrence networks of gut bacteria communities: Gut bacterial network diagrams are shown for four groups of frogs: FH (a), FNH (b), MH (c), MNH (d). Networks are based on pairwise Spearman’s correlations between abundant taxa (relative abundances of ASV > 0.01%). Each indicated connection has a correlation coefficient > |0.9| and a P-value < 0.01. Node size is proportional to relative abundance. The network for frog intestine samples with ASVs coloured by modularity

To elucidate the interactions between frog gut microbes in relation to sex and health status, four microbial interaction networks were constructed (Fig. 4), focusing on large modules in networks with at least 10 nodes through modularity. The diverse topological features of the four networks indicated that the co-occurrence patterns of microbes differed considerably across sexes and health states. The total number of nodes varied slightly (from 180 to 196), but the total number of links ranged from 440 (MH) to 1251 (MNH) (Table S1). Moreover, the number of nodes, total links, network density, average degree, and number of modules were strikingly similar in both female frog groups, with their network structures also closely related, exhibiting relatively high complexity and modularity (0.84–0.897). In male frogs, however, the disease group displayed much higher topological parameters than the healthy group, and their microbial communities exhibited the most complex networks with the highest relative modularity values (0.911) and modules (12); MH had the simplest of all networks and the lowest degree of modularity (0.817), with only four large modules. Surprisingly, we found that among frogs of the same health status, network complexity differed greatly between females and males. Specifically, with females being more complex than males within the H group, whereas males displayed greater complexity than females within the NH group.

**Fig. 5:**
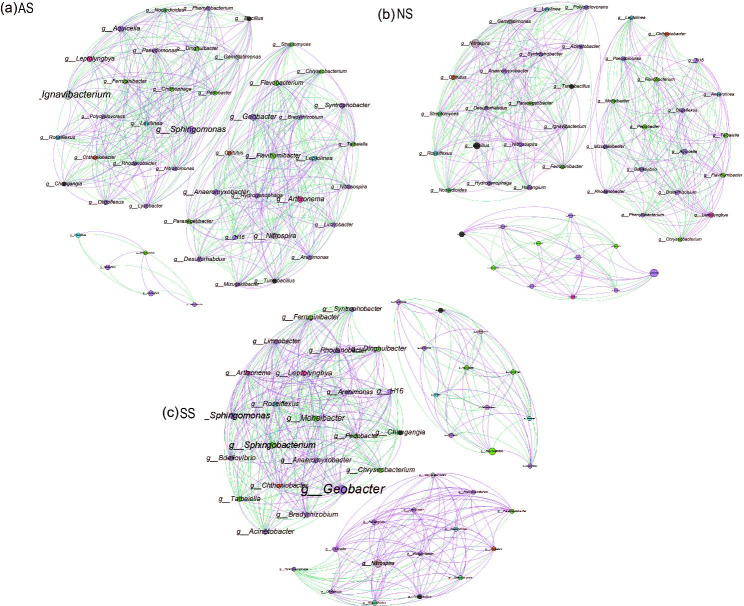
Bacterial co-occurrence networks in frog habitat soils: Network diagrams are generated for AS(a), NS(b), and SS(c) based on correlation analysis. Connections represent strong (Spearman’s P > 0.9) and significant (P value < 0.01) correlations for different habitat soils. Node names denote taxa at the bacterial genus level, with node size proportional to the number of links (degree) and colored by bacterial phyla. Networks consist of closely related bacterial modules to identify keystone taxa (module hubs), with node size proportional to relative abundance. Purple edges indicate positive interactions, while green edges signify negative interactions

To further investigate the interactions between bacterial communities in different frog habitat soils, we constructed a Spearman correlation-based network for the top 50 genera (Fig. 5) and analyzed the fundamental topological properties of the networks (Table S2). AS had the highest number of nodes, total connections, positive connections, and average degree of network density among all soils, with values of 50, 472, 51.27%, 0.401, and 19.265, respectively, indicating that the bacterial network of AS was the most complex and the positive associations between genera were intense. The lowest number of total connections, negative connections, and network density were found in NS soil, indicating that NS had the simplest networks. Further modularity analysis revealed the same significant modules for all three soils, with SS having the highest modularity (0.558) in all networks. In the AS network, *Ignavibacterium* (p__Ignavibacteriae) and *Sphingomonas* (p__Proteobacteria) were detected as the key taxa (strongest interaction). In the NS network, *g_Nitrosospira* (p__Nitrospirae) and *Bacillus* (p__Firmicutes) were similarly designated as key taxa in most genera in the third module, but they were much less closely related than in other soil network structures. The relatively more key taxa in SS are *Geobacter* (p__Proteobacteria), *Sphingomonas* (p__Proteobacteria), *Sphingobacterium* (p__Bacteroidetes), and *Bradyrhizobium* (p__Proteobacteria). Additionally, we found that these key taxa were primarily significantly and positively correlated with other genera.

### Prediction of bacterial metabolic function variation based on PICRUSt and FAPROTAX

**Fig. 6:**
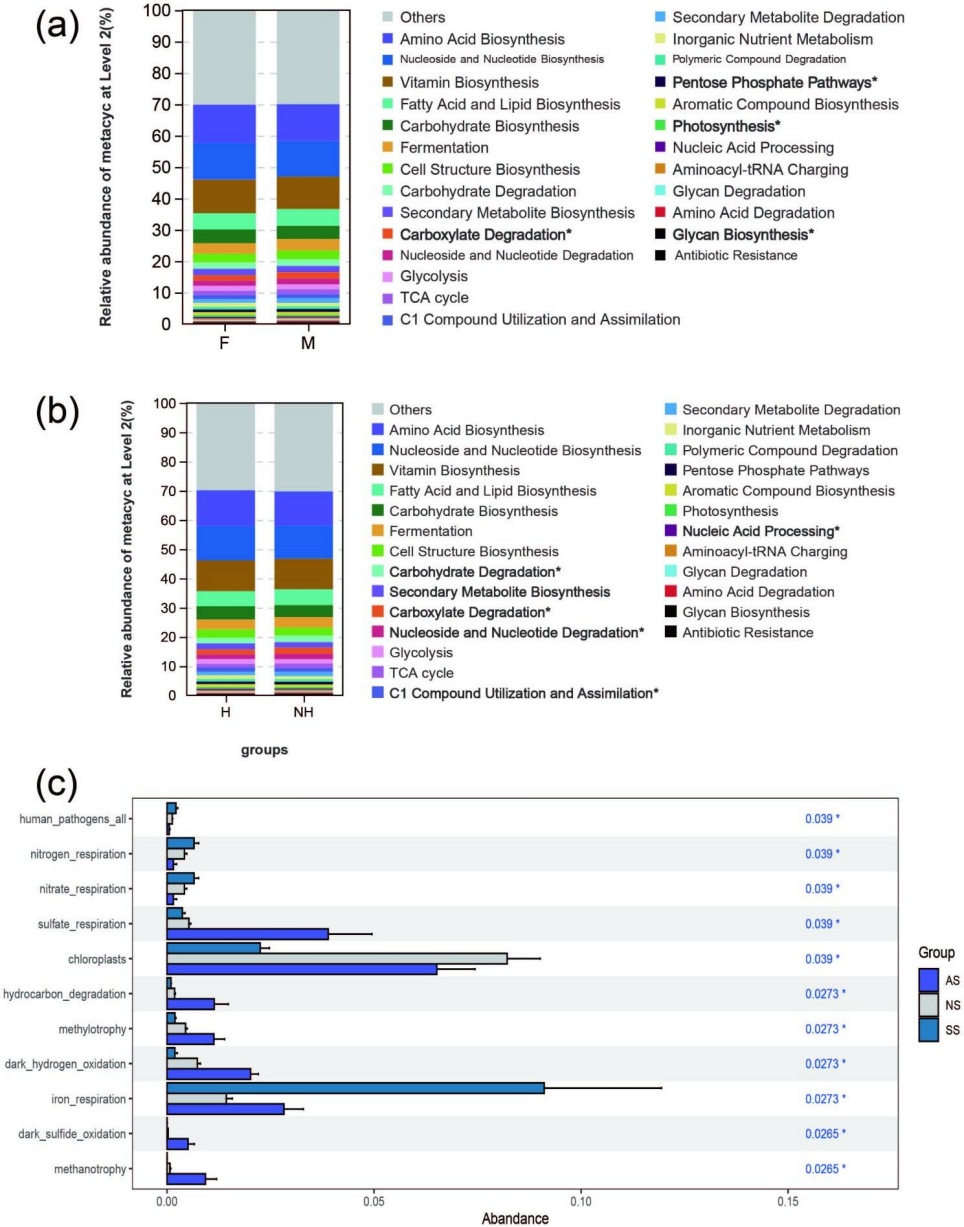
Predicted gene relative abundances in 16S rRNA amplicons: **(a, b)** Top 26 (> 0.01%) functional groups predicted by PICRUSt based on MetaCyc databases at the second level. **(c)** Bacterial functional groups from the FAPROTAX database to define habitat differences in soil microbiota using Kruskal-Wallis rank sum tests, with * indicating significance at 0.05 level

As the analyses above demonstrated that gut microbiota were differentiated by sex and health status, we investigated whether these gut bacteria function differentially in metabolism or physiology of frogs. We were able to assign 417 out of 1,214 bacterial ASVs (34.35%) predicted by PICRUSt. Furthermore, we predicted 42 functional groups in MetaCyc at the second level, with functional bacteria of Vitamin Biosynthesis, Amino Acid Biosynthesis, Nucleoside and Nucleotide Biosynthesis, Fatty Acid and Lipid Biosynthesis, and Carbohydrate Biosynthesis being dominant in the gut of frogs. The relative abundances of Carboxylate Degradation, Pentose Phosphate Pathways, and Glycan Degradation were significantly higher in the M group than the F group, while Photosynthesis was significantly higher in the F group than the M group (Fig. 6a). Considering the health state of the frog, Carbohydrate Biosynthesis, Secondary Metabolite Biosynthesis, C1 Compound Utilization and Assimilation, and Nucleic Acid Processing had significantly higher relative abundances in H than the NH group, but Nucleoside, Nucleotide Degradation and Carboxylate Degradation were significantly lower in the H group than in the NH group (Fig. 6b).

FAPROTAX enables the analysis of biogeochemical cycling processes in environmental samples like soil, particularly for the functional annotation prediction of elemental cycles such as carbon, phosphorus, sulfur, and nitrogen. Fifty major function groups were obtained based on the FAPROTAX tool, and only 1,588 ASVs were identified as known functions, representing 35.6% of the total ASVs (4,461). There were 11 functions that differed significantly between soil habitats, for example, human_pathogens_all, nitrogen_respiration, and nitrate_respiration; most notably, human_pathogens_all, nitrogen_respiration, nitrate_respiration, nitrogen_fixation, and iron_respiration were significantly enriched in SS compared to the others. On the other hand, sulfur_respiration, hydrocarbon_degradation, methylotrophy, and dark_sulfur oxidation were significantly higher in AS than in the others. Interestingly, chloroplasts were the only function found in NS that was significantly greater than in other frog-inhabiting soils (Fig. 6c).

### Bacterial community assembly patterns in frog gut and soils

**Fig. 7:**
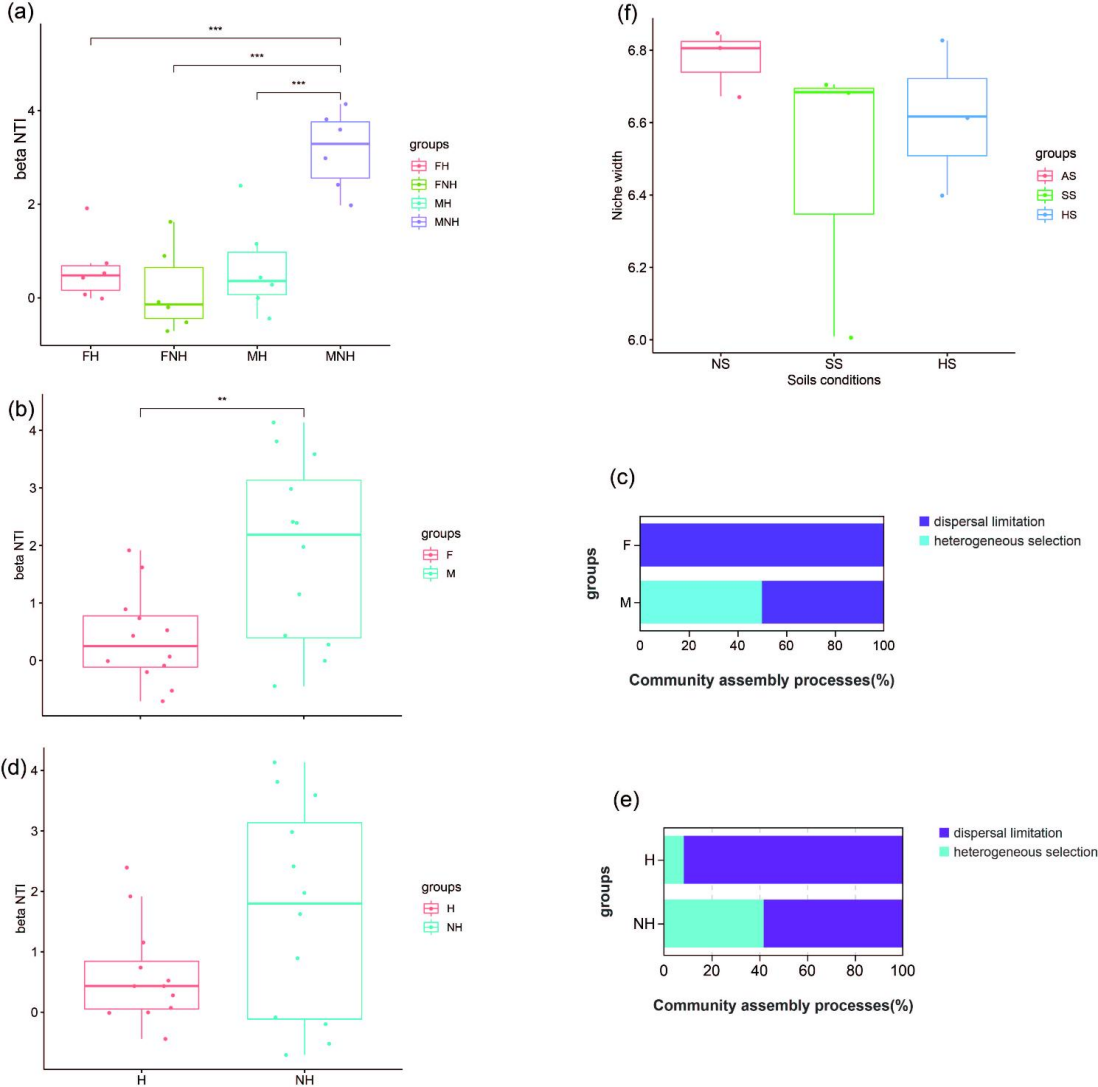
Bacterial community assembly patterns: **(a, b, d)** b-Nearest Taxon Index (bNTI) of bacterial communities in all frogs, with horizontal dashed lines (bNTI values at 2 and − 2) indicating significance thresholds. **(c)** Bray-Curtis-based Raup-Crick (RCbray) values of frog bacterial communities between F and M groups, with horizontal dashed lines at 0.95 and − 0.95. Community assembly turnover is driven by various deterministic processes, including heterogeneous selection, and stochastic processes, including dispersal limitation by RCbray. **(e)** Comparisons between F and M groups, and H and NH groups. Boxplots depict the mean niche width **(f)** comparison of frog-associated soil bacteria

To further explore the relative contribution of stochastic and deterministic processes to bacterial community assembly, bNTI was calculated based on OTU abundance and its phylogenetic distance. The average bNTI value (3.15) in MNH was remarkably higher than the others, indicating that the deterministic processes are more important for community assembly than the stochastic processes in MNH; the other frog group had the opposite result (Fig. 7a). The average bNTI value (2.17) in the M group was significantly higher than that in the F group (0.21) (Fig. 7b), and the RCbray values showed that heterogeneous selection and dispersal limitation are equally important in the deterministic processes in the M group. All bNTI values in the F group were lower than 2 and higher than − 2, and all RCbray values were higher than 0.95, indicating that heterogeneous selection of stochastic processes determines the assembly of gut bacteria in the F group (Fig. 7c). However, there was no significant difference in the average bNTI between H and NH groups, and the stochastic processes dominate in the assembly of the bacterial community (Fig. 7d). Furthermore, the majority (91.7%) of H group belonged to dispersal limitation in community assembly, and dispersal limitation (58.3%) were also slightly more important than heterogeneous selection (41.7%) in the NH group (Fig. 7e). The value of the niche width index for frog-associated soil bacteria was NS > SS > AS (Fig. 7f), suggesting that deterministic processes had a greater influence on community assembly in SS.

## Discussion

### Effects of sex on the gut microbiota of frogs

In this study, we collected frogs(*P. nigromaculata*)of different sexes from the same habitat and with similar dietary composition in southern China to explore the relationship between gut microbial communities and their host. In terms of alpha diversity, we did not find any significant differences in bacterial diversity and abundance indices between sexes in frog intestinal samples. Diversity within the gut microbiota is influenced by many factors. For example, urbanization has increased the diversity of commensal microbes residing in the gut and skin, but at the expense of community stability [11]. However, the comparison analysis of the gut microbiota of three species of Anura frogs from mountain streams, conducted by Zhuo Chen, found no significant differences in alpha diversity indices between the different frog species [48]. Studies on the influence of sex on frog gut microbial diversity are scarce. Yilin Shu’s study revealed no differences in intestinal microbes diversity between male and female healthy Chinese concave-eared frogs (*Odorrana tormota*) [17]. This is in line with our results, which suggest that sex is not a key factor influencing the diversity of α-factors in the frog gut.

However, beta diversity analysis indicated that bacterial composition in frogs was significantly different between sexes. Previous studies have consistently shown that factors such as frog species [48], sex of *O. tormota* [17], and developmental stage [6] significantly affect gut bacterial composition. 16S rRNA sequencing and metagenomic studies confirmed that Firmicutes, Bacteroidetes, and Proteobacteria are enriched in various frog gut microbiomes [49, 50], with no significant differences in relative abundance between sexes. Our findings indicate that Firmicutes, Bacteroidetes, Verrucomicrobia, and Proteobacteria are dominant phyla in frog gastrointestinal tracts with no differences in abundance. These results are consistent with previous findings that animals living in similar environments and under similar predation conditions tend to have similar microbial taxa at higher taxonomic levels [51]. Current research suggests that significant differences in gut microbial composition between sexes are observed at some lower taxonomic levels [52]. These differences may be due to subtle variations in predation between the sexes [15, 53]. At the genus level, we found that frog intestines had a significantly higher abundance of *Ruminococcaceae* in the female frog, whereas *Laribacter* was significantly enriched in the male frog. *Alistipes*, the Gram-negative bacterium, may have protective effects against liver fibrosis, cancer immunotherapy, and cardiovascular diseases, and is also associated with colorectal cancer and depression [15]. Yilin Shu’s study found that *Robinsoniella* was significantly more abundant in female frogs than in males [17]; however, *Alistipes* was more abundant in males, potentially benefiting frog health. Differences in a few bacterial taxa may be due to the subtle variations in predation between sexes.

Network complexity plays a crucial role in understanding microbial interactions [54]. Francis constructed interaction networks (symbiosis and competition) of tree frog gut microbiota, such as the symbiotic relationship between *Garvieae* and *Corynebacterium variabile* [55]. Our results revealed that differences in health status can distinctly alter co-occurrence networks of the frog gut microbiota between sexes. Specifically, in healthy frogs, females exhibited greater bacterial network complexity and modularity compared to males, which may be attributed to the regulation of symbiotic and competitive relationships among microbial communities. Interestingly, this effect was reversed in unhealthy frogs, with males having the most complex bacterial networks. Moreover, a study by Liangliang and colleagues found that seasonal changes reduced the complexity of frog (skin and gut) microbiota networks from summer to autumn [12]. A previous study showed that high network complexity implies the need for a more stable microbial network to withstand harmful bacterial interference from the environment [56]. Consequently, we hypothesise that in healthy frogs, the microbial network of females may be more resistant to environmental disturbances than that of males; whereas MID-infected males still show the strongest environmental resistance.

We discovered that sex influences the community assembly mechanisms of intestinal bacteria in frogs. Overall, deterministic processes were the dominant factors driving the assembly of symbiotic bacterial communities in male frogs. However, heterogeneous selection (selection caused by varying conditions) of stochastic processes determines the assembly of intestinal bacteria in the female frog, leading to higher phylogenetic composition variations. There is a strong connection between bacterial phylogeny and function, so functional predictions can provide useful insights for the vast uncultured microbial communities obtained from amplicon sequencing [57]. Based on PICRUSt, the most abundant functions in Level 2 MetaCyc metabolic pathways include Vitamin Biosynthesis and Amino Acid Biosynthesis, mainly associated with biosynthesis. Existing research comparing the gut microbiota of three frog species found that the most abundant gene functions within these communities are primarily related to metabolism, specifically amino acid metabolism, carbohydrate metabolism, and metabolism of cofactors and vitamins [48]. This finding differs significantly from our results. Additionally, Yilin Shu’s research indicates that the COG functional profile of frog gut metagenomes reveals a rich array of carbohydrate transport and metabolic pathways [17]. Interestingly, our study discovered that Glycan Degradation is more abundant in male frogs compared to females, which is related to carbohydrate metabolism. Thus, it can be inferred that the gene function differences in frog gut microbiota may be associated with dietary habits caused by sex differences.

### Changes in gut bacterial communities in response to health status of frogs

In humans, there have been numerous reports on the association between diseases and gut microbiota. For example, non-alcoholic fatty liver disease (NAFLD) reduces the diversity of gut microbiota, both in terms of α and β diversity [58]. Consequently, gut microbiota may reflect the immune system status and overall health of the host species [59]. Wengang’s study revealed that the richness, evenness, and abundance of microbial communities in the oral cavity and intestines of diseased bullfrogs were significantly higher than those in healthy bullfrogs. Furthermore, the abundance of *Elizabethkingia* markedly increased, while that of *Lactococcus* significantly decreased [23].

In this study, we examined the association between cataract infection and gut microbiota diversity and composition in frogs. Similarly, we found no significant differences in the richness and diversity of gut microbiota among frogs with different health statuses. Regarding microbial composition, we found that *Parabacteroides* was significantly enriched in the NH frog, while *Odoribacter* and *Akkermansia* were significantly enriched in the H frog. Yilin Shu’s research found that the phylum Actinobacteria was more abundant in infected individuals, and the genus *Akkermansia* was more abundant in healthy frogs [59]. This is consistent with our findings, indicating that diseases lead to an increase in these taxa. The presence of *Odoribacter* is closely related to host health and participates in the metabolism of carbohydrates, lipids, and amino acids. Its abundance may be altered in some obesity and inflammatory bowel diseases [60]. Therefore, we can infer that cataract disease may also suppress *Odoribacter*, which helps maintain the balance of gut microbial communities.

Stochastic processes dominate the assembly of bacterial communities in frogs with different health statuses, primarily influenced by dispersal limitation. These results suggest that these frogs have a lower dispersal rate, so disease may restrict the spread of symbiotic bacterial communities. Heterogeneous selection is the second factor after dispersal. Thus, varying environmental selection pressures in amphibians (different infection levels) may cause significant differences in frog gut community assembly to adapt to different environmental factors or selection pressures [61]. Wengang’s study suggests that pathogen (MID) infection may cause a decline in host immune function [23]. Consistently, our findings reveal that the majority of gut microbiota functions (especially Carbohydrate Biosynthesis and Secondary Metabolite Biosynthesis) are significantly stronger in healthy frogs compared to diseased ones.

### Environmental effects on soil bacterial community composition in frog habitats

In the above section, we investigated bacterial diversity in frog intestines; however, soil microbiota, which also share a symbiotic relationship with frogs, are also worth exploring. First, we found that the number of ASVs for soil bacteria in frog habitats was twice that of gut bacteria, suggesting that the diversity and richness of soil bacteria is significantly greater than that of gut bacteria. Among different habitats, bacterial community richness was significantly higher in NS than in AS and SS, but soil bacterial diversity did not differ significantly. Xiaomei Yi et al. conducted the first comprehensive study on the structure and function of soil microbial communities in rice field (RF) and showed that RF significantly increased the diversity and richness of bacterial and fungal communities [27]. This may be due to the increase in frog feces with increasing cultivation time, which favors the growth of various microorganisms. Studies on soybean soil have also reported that bacterial species richness is significantly higher in agricultural soil than in native soil, but diversity does not differ significantly between the two soil types [25]. In our study, we reached a conclusion contrary to the two aforementioned studies, possibly because the intense activity of frogs in HS and SS suppressed soil bacterial richness.

Further research indicated that the type of frog habitat had a significant effect on soil bacterial β-diversity. Interestingly, a study by Pérez-Jaramillo et al. showed that there were significant differences between soybean agricultural soil and native soil [25]. We observed that the dominant phyla of bacteria in frog habitat soil were mainly Proteobacteria, Bacteroidetes, Acidobacteria, and Chloroflexi, among which Firmicutes were most abundant in NS; Ignavibacteriae were much higher in AS than in other soils. However, their differences at the phylum level were not significant. The research of Xiaomei Yi et al. found that Proteobacteria, Acidobacteria, and Chloroflexi were the dominant bacterial communities in rice-frog cultivation (RF) soil, and the specific bacterial taxa enriched in RF played an indispensable role in organic matter decomposition and soil C, N, and P transformation processes [27]. They were also identified in our samples. In addition, research has shown that Acidobacteria have a higher relative abundance in native soil than in soybean agricultural soil [25], and in our study, Acidobacteria were found to be the highest in SS (63.28%). Acidobacteria are generally considered to be oligotrophic, acidic bacterial species in soil [62], and their diversity in metabolic characteristics renders them potentially important communities in soil nutrient cycling [63]. This may be attributed to the fact that frog culture promotes the enrichment of Acidobacteria in soybean soil. At the genus level, we observed significant differences in the dominant genera *Bacillus*, *Nitrosospira*, and *Geobacter*. Another study found that the core bacterial genera in agricultural soybean soil include *Rhizobium*, *Bradyrhizobium, Mesorhizobium*, and *Sphingomonas*, with a considerable portion comprised of nitrogen-fixing bacteria [25]. In our study, *Sphingomonas* and *Bradyrhizobium* were significantly higher in abundance in soybean soil compared to other soil types, suggesting that soybean soil in frog farming is rich in nitrogen-fixing bacteria.

Analysis of bacterial networks in frog habitat soils revealed an unprecedented complexity of the HS network, due to nutrient inputs from frog excreta and food remains. Pivotal taxa, Ignavibacterium and Sphingomonas, were identified as essential for the stability of the HS network. In contrast, the SS exhibited unique modularity and hosted key taxa such as Geobacter and Sphingomonas, which were positively correlated with other genera. Notably, while native soil promote complex bacterial interactions, soybean soil facilitate the establishment of nutrient-rich organisms [25]. We therefore propose that soybean soil in frog farming inherently shape the modularity of bacterial networks, allowing critical bacterial taxa to establish efficiently.

Furthermore, we constructed bacterial ecological networks in frog habitat soils. This research revealed that the HS bacterial network structure was the most complex, with the tightest connections between bacteria. As the soil with the most active frog activity, this may be due to frog excreta and food providing a nutrient source for soil microbes. *Ignavibacterium* and *Sphingomonas* were detected as the most critical taxa (strongest interaction) in HS, with the stability and construction of the bacterial network structure primarily relying on them. In terms of bacterial community modularity, SS exhibited the most distinct pattern, while also possessing the highest number of key taxa, including *Geobacter*, *Sphingomonasa*, *Sphingobacterium*, and *Bradyrhizobium*. These key taxa displayed a significantly positive correlation with other genera. Previous research found that the interactions between bacterial taxa in native soil environments were more complex than those in soybean agricultural soil, but soybean soil favors the establishment of nutrient-rich organisms [25]. Based on these findings, we therefore propose that soybean soil in frog farming inherently shape the modularity of bacterial networks, allowing critical bacterial taxa (such as *Geobacter, Sphingomonas*, *Sphingobacterium*, and *Bradyrhizobium*) to establish efficiently.

Bacteria play a crucial role in soil nutrient cycling, and their functions determine soil fertility and microbial vitality to some extent [64]. According to FAPROTAX, we predicted that 35.6% of ASVs possess potential ecological functions. Most functions in soybean soil (SS) were significantly higher than in the other two soil types, such as nitrogen_respiration, iron_respiration, sulfur_respiration, and nitrogen_fixation. Iron_respiration was notably higher in SS than in other soils, which might be closely related to the presence of abundant nitrogen-fixing bacteria in that environment. Furthermore, we found that many functions were significantly higher in HS than in the other two soil types, including hydrocarbon_degradation, dark_hydrogen_oxidation, methanotrophy, methylotrophy, and dark_sulfide_oxidation. HS provides favorable conditions for bacteria associated with dark_hydrogen_oxidation and dark_sulfide_oxidation due to its moist and light-avoiding environment. In contrast, NS areas lack interference from plants and animals such as frogs, resulting in generally lower functional microorganisms and weaker soil microbial metabolic activity compared to other soils. Regarding the assembly and ecological niche width of soil bacterial communities, we applied the neutral model to fit bacterial communities in different frog habitats. We found that the dispersal limitation of NS bacterial communities was much more severe. NS is considered to be heterogeneous and discontinuous soil for microbes [65]. In contrast, due to the influence of soybean plants (SS) or frog habitation (AS), the soil environment is more uniform. As a result, more ecological niches exist in NS soil. Additionally, deterministic mechanisms had a more significant impact on the community composition of SS, likely because deterministic processes have a more substantial influence on soybean soil with a narrower niche width.

## Conclusions

Our study illuminated the important role of sex, health status, and habitat in shaping the composition and assembly processes of symbiotic bacteria among frogs. Key findings indicated that sex and health had a strong influence on gut bacterial taxonomy at the genus level. Healthy frogs had a more robust gut microbiome than those suffering from meningitis-like infectious disease (MID). In particular, the microbial network structure was more complex in healthy female frogs than in males, with diseased males showing the greatest complexity. In male frogs, the assembly of gut bacteria was predominantly deterministic, while in females it was largely stochastic. Regarding the influence of symbiotic bacteria in frog habitat soils (frog aggregation, native and soybean soils), they had significant effects on bacterial diversity. For instance, soybean soil had higher pathogens and nitrogen functions, whereas frog-aggregation soil had an increased capacity for sulfur respiration and hydrocarbon degradation. These findings have important implications for sustainable frog farming and ecosystem management. They highlight the complex relationship between frog-associated microbiota and their environment and suggest that manipulation of these factors could improve agricultural productivity and ecological balance. However, limitations of our study remained, particularly in understanding the complex interactions between host immune responses, environmental factors, and specific bacterial taxa. Future research will need to focus on these areas to gain a more comprehensive understanding of how these elements interact to influence frog health and habitat.

### Electronic supplementary material

Below is the link to the electronic supplementary material.

Supplementary Material 1

Supplementary Material 2

## Data Availability

The datasets used and/or analysed during the current study are available from the corresponding author on reasonable request. The raw amplicon data have been submitted to NCBI GenBank (accession number: PRJNA1023626).
