# Supplementary material for: Sex, health status and habitat alter the community composition and assembly processes of symbiotic bacteria in captive frogs
Source: BMC Microbiol. 2024 Jan 23;24:34. doi: 10.1186/s12866-023-03150-y (PMC10804495; doi:10.1186/s12866-023-03150-y)
Supplement: Supplementary file 1 — Supplementary Material 1 [file 12866_2023_3150_MOESM1_ESM.doc]

**
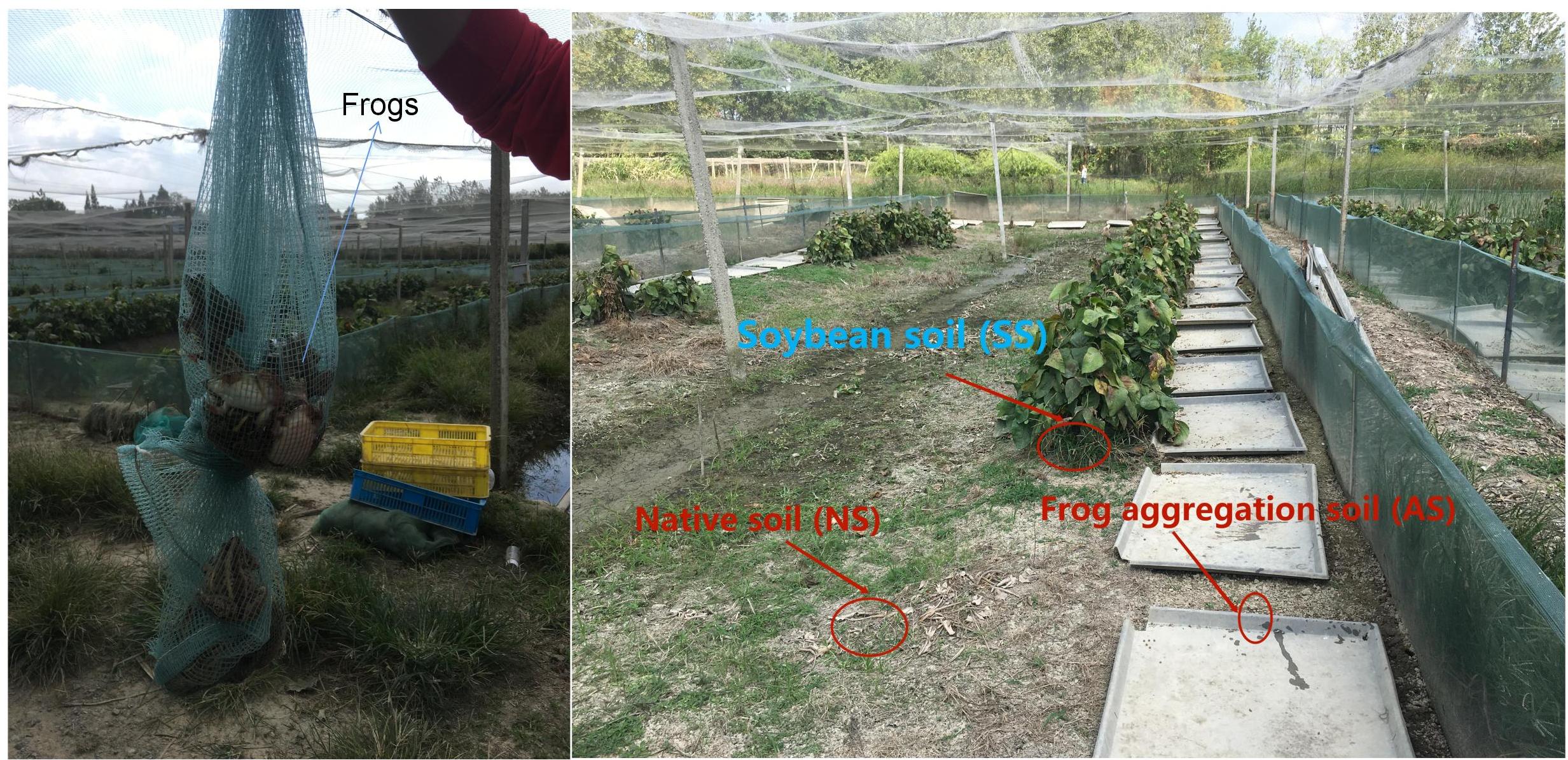
**

## **Fig. S1** Experimental site and sample collection from the frog farm


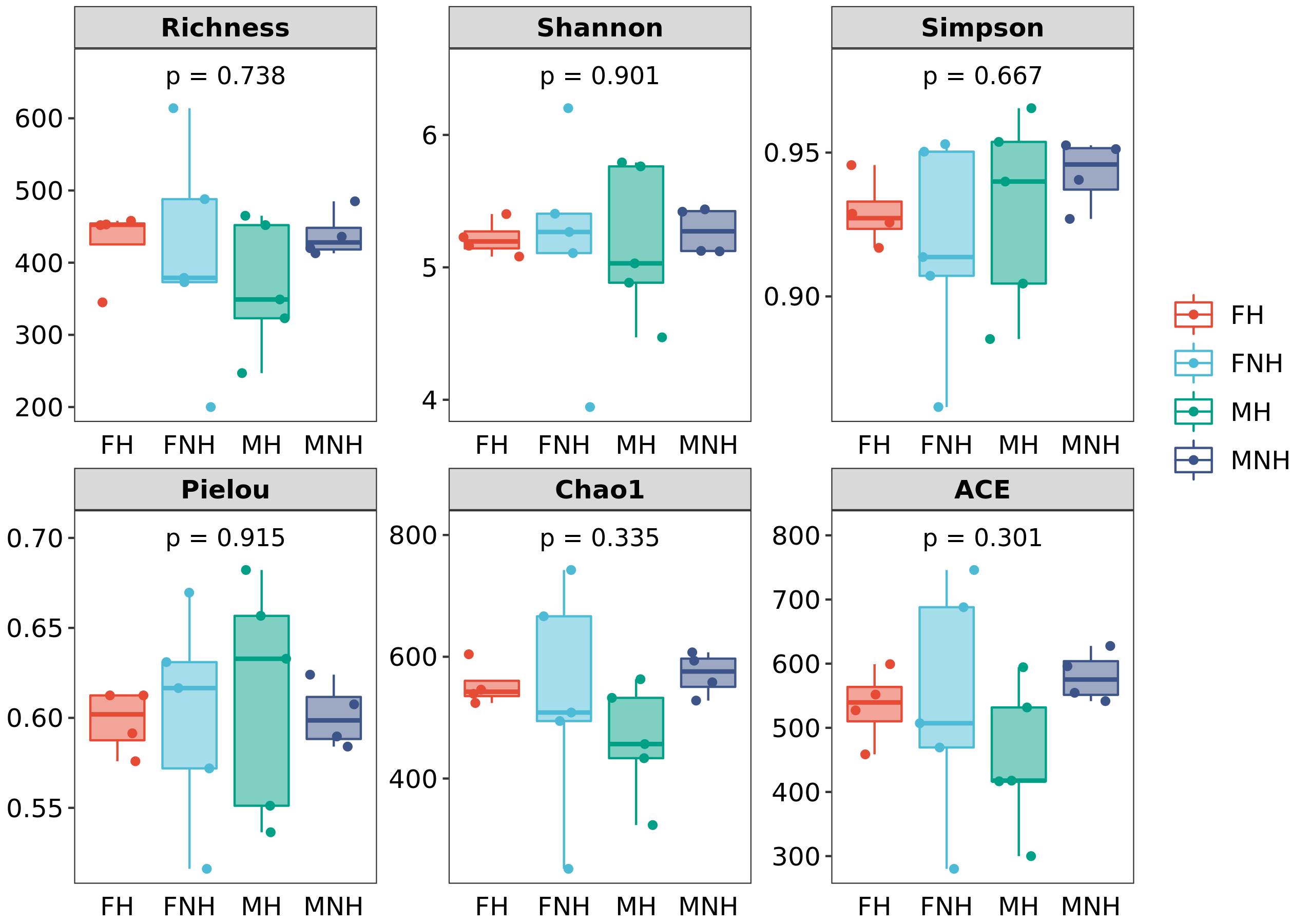


**Fig. S2** Comparative analysis of the alpha diversity index of bacterial 16S rRNA gene in different frogs:(a)observed species(ASVs), (b) Chao,(c)ace,(d) Shannon, (e) Simpson and (f) Simpson were calculated by four treatments. Statistically important differences were determined by one-way ANOVA (P < 0.05).


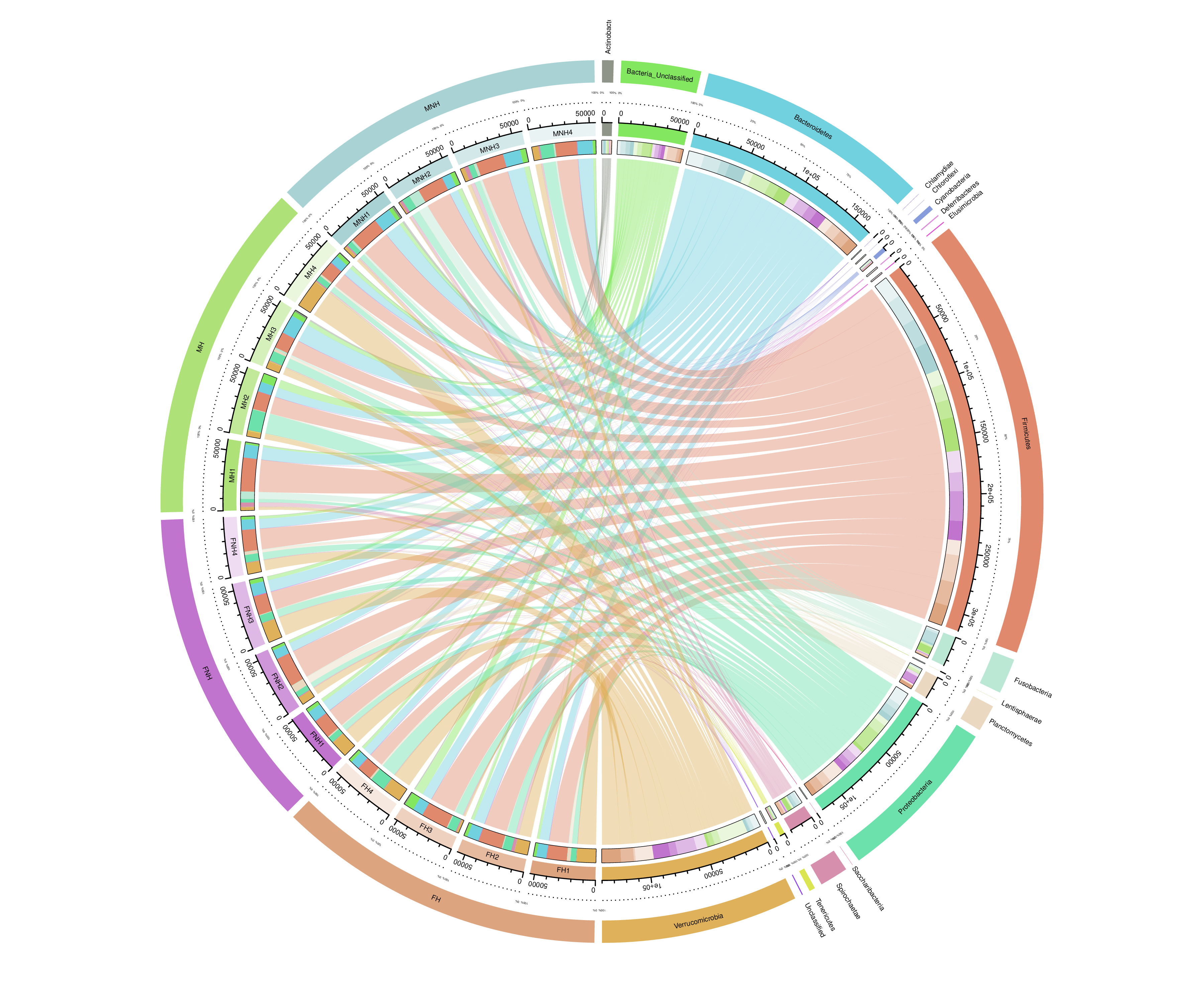


**Fig. S3** CIRCOS plots of total phylum of gut bacteria in each frog sample, showing the relative abundance among the phyla.


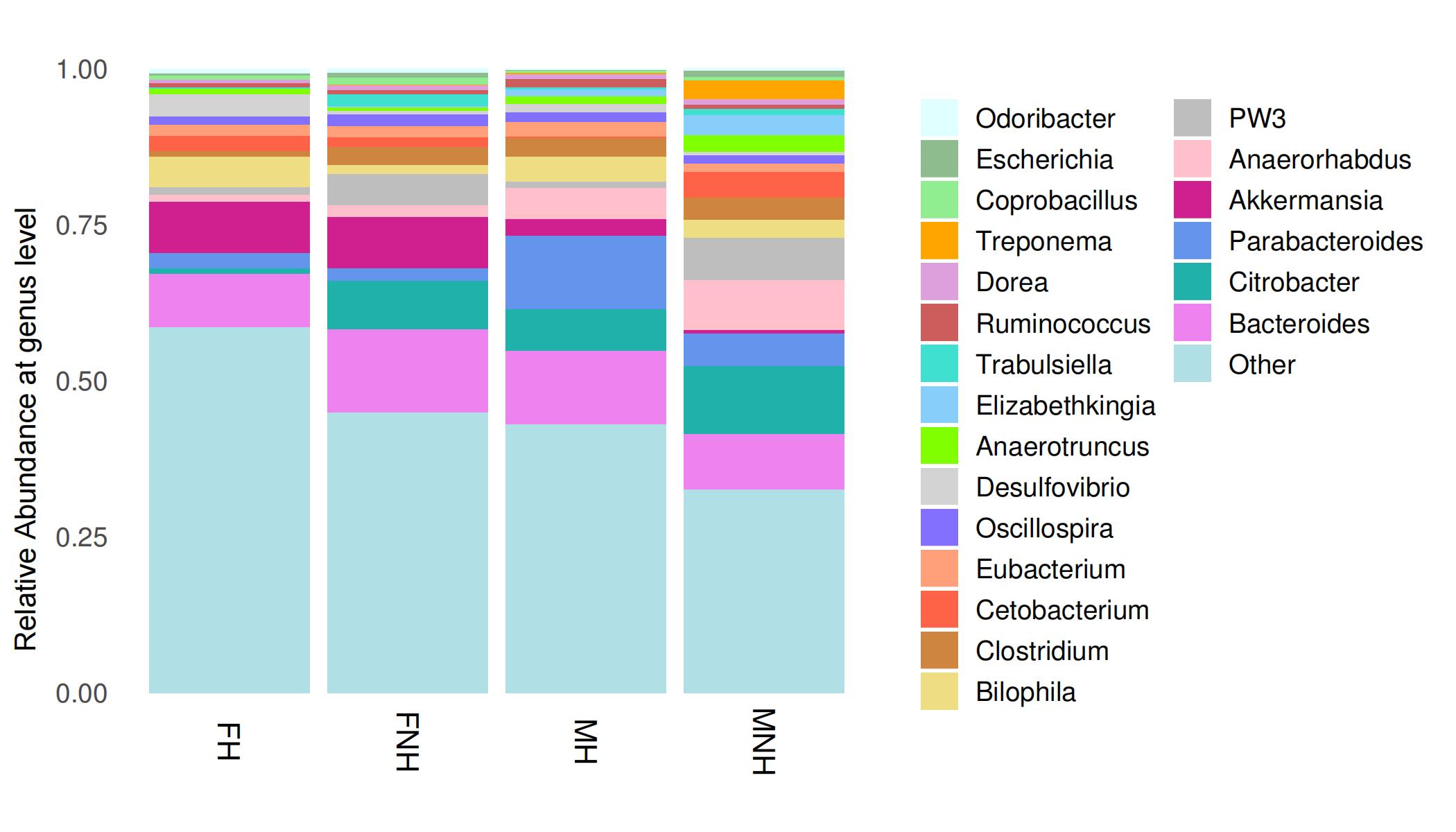


**Fig. S4** Barplot of relative abundance of dominant genera in gut bacteria of frogs.


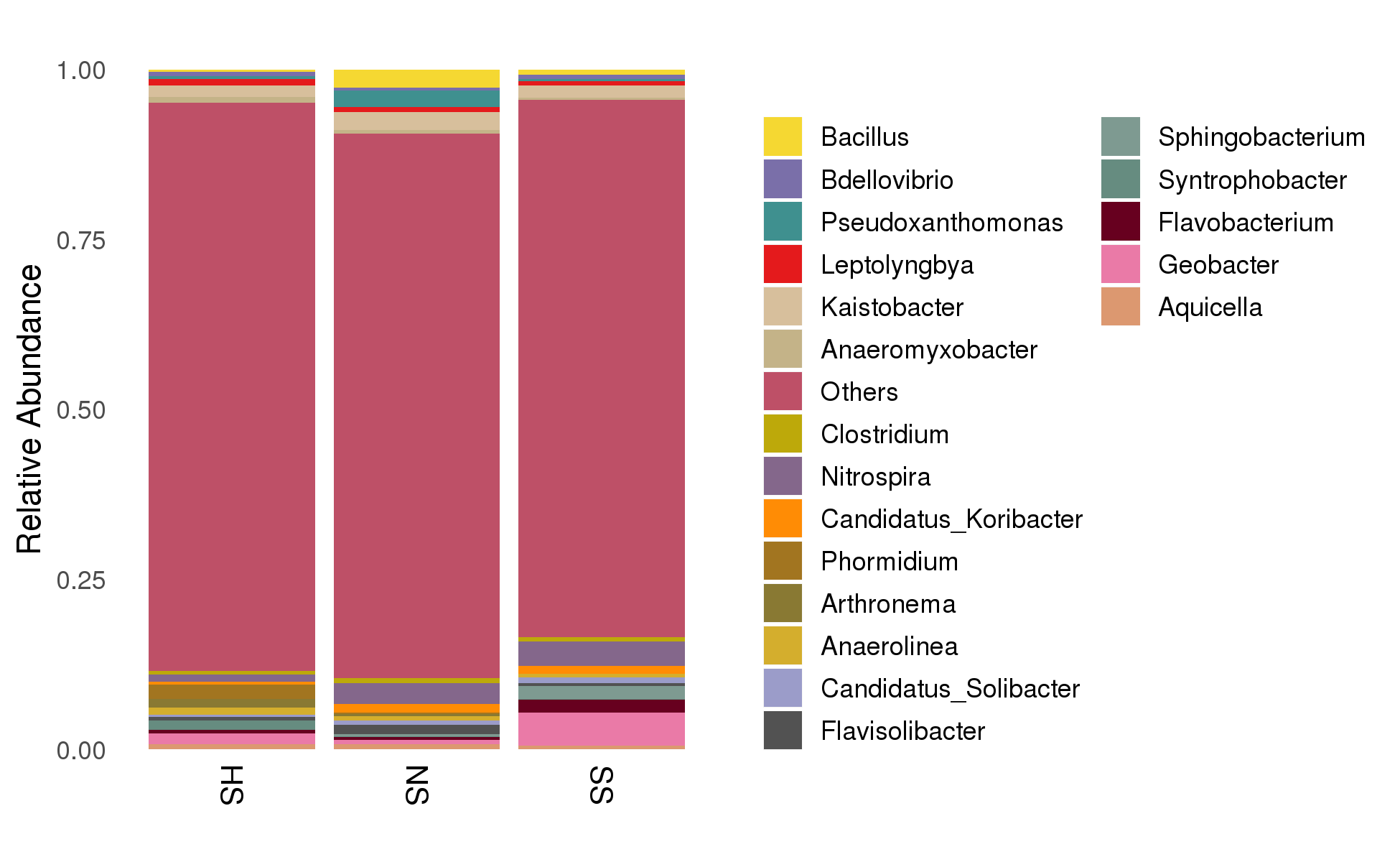


**Fig. S5** Barplot of relative abundance of dominant genera of soil bacteria in frog habitats (AS, NS, SS).

Table S1 Topological properties of the correlation network diagram for the gut bacterial community at the OTU level.

| Parameters | FH | FNH | MH | MNH |
| --- | --- | --- | --- | --- |
| nodes | 180 | 186 | 185 | 196 |
| Total links | 583 | 627 | 440 | 1251 |
| Positive links | 29 (61.23%) | 42 | 47 | 50.73% |
| Negative links | 226 (38.77%) | 208 | 137 | 608 49.27% |
| Clustering coefficient | 0.969 | 0.951 | 0.907 | 1 |
| Network density | 0.036 | 0.036 | 0.022 | 0.063 |
| Network diameter | 4 | 6 | 6 | 6.5 |
| Average degree | 6.478 | 6.783 | 4.35 | 12.51 |
| Modularity | 0.897 | 0.84 | 0.817 | 0.911 |
| No. of modules | 8 | 6 | 4 | 12 |
| No. of large modules | 7 | 7 | 4 | 4 |
| No. of nodes in large modules | 137 | 190 | 281 | 162 |
| Percentages of nodes in large modules** (%) | 83.53 | 82.61 | 84.90 | 94.74 |
| R square of power-law | 0.821 | 0.905 | 0.681 | 0.730 |

**Table S2** Topological properties of a network diagram for the soil microbiome at the genus level in frog habitats.

| Parameters | HS | NS | SS |
| --- | --- | --- | --- |
| nodes | 49 | 50 | 50 |
| Total links | 472 | 416 | 424 |
| Positive links | 51% | 50% | 62% |
| Negative links | 48% | 50% | 38% |
| Network density | 0.401 | 0.34 | 0.346 |
| Average degree | 18.88 | 16.64 | 16.96 |
| Modularity | 0.521 | 0.605 | 0.558 |
| No. of modules | 3 | 3 | 3 |
| R square of power-law | 0.821 | 0.681 | 0.730 |
